# Insecticide Resistance in the Dengue Vector *Aedes aegypti* from Martinique: Distribution, Mechanisms and Relations with Environmental Factors

**DOI:** 10.1371/journal.pone.0030989

**Published:** 2012-02-21

**Authors:** Sébastien Marcombe, Romain Blanc Mathieu, Nicolas Pocquet, Muhammad-Asam Riaz, Rodolphe Poupardin, Serge Sélior, Frédéric Darriet, Stéphane Reynaud, André Yébakima, Vincent Corbel, Jean-Philippe David, Fabrice Chandre

**Affiliations:** 1 Unité Mixte de Recherche MIVEGEC (UM1-CNRS 5290-IRD 224), Institut de Recherche pour le Développement (IRD), Montpellier, France; 2 Laboratoire d'Ecologie Alpine (LECA), UMR 5553 CNRS-Université de Grenoble, Grenoble, France; 3 Centre de la Démoustication, Conseil General de la Martinique, Martinique, France; 4 Centre de Recherche Entomologique de Cotonou (CREC), Institut de Recherche pour le Développement (IRD), Cotonou, Benin; New Mexico State University, United States of America

## Abstract

Dengue is an important mosquito borne viral disease in Martinique Island (French West Indies). The viruses responsible for dengue are transmitted by *Aedes aegypti*, an indoor day-biting mosquito. The most effective proven method for disease prevention has been by vector control by various chemical or biological means. Unfortunately insecticide resistance has already been observed on the Island and recently showed to significantly reduce the efficacy of vector control interventions. In this study, we investigated the distribution of resistance and the underlying mechanisms in nine *Ae. aegypti* populations. Statistical multifactorial approach was used to investigate the correlations between insecticide resistance levels, associated mechanisms and environmental factors characterizing the mosquito populations. Bioassays revealed high levels of resistance to temephos and deltamethrin and susceptibility to *Bti* in the 9 populations tested. Biochemical assays showed elevated detoxification enzyme activities of monooxygenases, carboxylesterases and glutathione S-tranferases in most of the populations. Molecular screening for common insecticide target-site mutations, revealed the presence of the “knock-down resistance” V1016I *Kdr* mutation at high frequency (>87%). Real time quantitative RT-PCR showed the potential involvement of several candidate detoxification genes in insecticide resistance. Principal Component Analysis (PCA) performed with variables characterizing *Ae. aegypti* from Martinique permitted to underline potential links existing between resistance distribution and other variables such as agriculture practices, vector control interventions and urbanization. Insecticide resistance is widespread but not homogeneously distributed across Martinique. The influence of environmental and operational factors on the evolution of the resistance and mechanisms are discussed.

## Introduction

Dengue fever is a major public health problem in Martinique Island (French-West-Indies) and occurs in an endemo-epidemic pattern [1]. The last decade saw a dramatic resurgence of the virus with 5 major dengue outbreaks and more than 111,000 reported cases in the island witch counts more than 400,000 inhabitants [2]. As there is still no specific treatment and efficient vaccine available, vector control against *Aedes aegypti* remains the most effective solution to prevent dengue transmission. Environmental management, educational programs and mechanic elimination of the breeding habitats are continuously implemented but currently, the use of chemical and biological agents are the main methods for reducing the incidence of the disease.

Unfortunately, vector control programs are facing operational challenges with the emergence and development of insecticide resistance in dengue vectors, especially *Ae. aegypti* [3]. In Martinique, resistance to organophosphates (OPs) and pyrethroids (PYRs) has been reported since the 1980s and 1990s respectively [4], [5], and this resistance has recently been shown to be negatively impacting on the efficacy of vector control interventions [6]. A molecular study conducted by Marcombe [7] showed the involvement of both metabolic and target site based resistance mechanisms in a wild population of Martinique (Vauclin) strongly resistant to OPs and PYRs. Biochemical assays revealed significant elevated activities of cytochrome P450 monooxygenases (P450s), glutathione S-transferases (GSTs) and carboxy/cholinesterases (CCEs) at both larval and adult stages. Microarray and quantitative RT-qPCR experiments showed a significant constitutive over-transcription of multiple detoxification genes at both larval and adult stages. Sequencing of the voltage-gated sodium channel showed high allelic frequency (71%) of the “knockdown resistance” (*Kdr*) mutation (V1016I) in this Martinique population which confers resistance to DDT and PYRs [8], [9].

The massive use of different insecticide families for vector control since the 1950s has probably contributed to select for insecticide resistance in mosquitoes [10]. In Martinique island, temephos (Abate®) was used for decades for larval control (abandoned in 2009 to respect the recent European biocide legislation; European Commission Environment Biocidal Products, 1998) and now *Bacillus thuringiensis var israelensis* (*Bti*, Vectobac®) is the only insecticide used for such application. Space spraying treatments with vehicle-mounted or portable thermal fogger (aerial or inside application, respectively) are implemented during inter-epidemic periods (i.e. when high entomological indices are reported) and during outbreaks to rapidly kill infected adult mosquitoes [11]. DDT and several OPs (e.g., malathion, fenitrothion) were used since the 1950s but there was a switch to PYRs in the early 1990s [5] because of their rapid knockdown action and low mammalian toxicity (WHO, 2006b). Currently, deltamethrin (K-Othrine 15/5®) and to a lesser extend synergized natural pyrethrins (AquaPy®) are used for the control of adult mosquitoes. In addition, Martinique is an island with intensive agriculture practice (mainly sugar cane and bananas) where huge amount of pesticides have been applied for crop protection. Pesticides used in agriculture include organochlorines (OCs), OPs and carbamates. Recently, Bocquené and Franco [12] reported the widespread contamination of rivers and soils in Martinique with pesticides and particularly with high levels of the OCs chlordecone and lindane. The constant exposure of the mosquito populations to these pesticides associated with the increasing urbanization may have led to the selection of particular detoxification genes and to an increased tolerance to pesticides [13], [14], [15], [16].

In this study we investigated the insecticide resistance level and the associated molecular mechanisms in nine *Ae. aegypti* populations collected in several ecological settings in Martinique island. A statistical multifactorial approach was adopted to investigate the possible relationship between resistance levels, associated mechanisms and environmental factors such as agriculture, pollution and urbanization in Martinique populations.

## Materials and Methods

### Mosquito strains and populations

**Figure 1:**
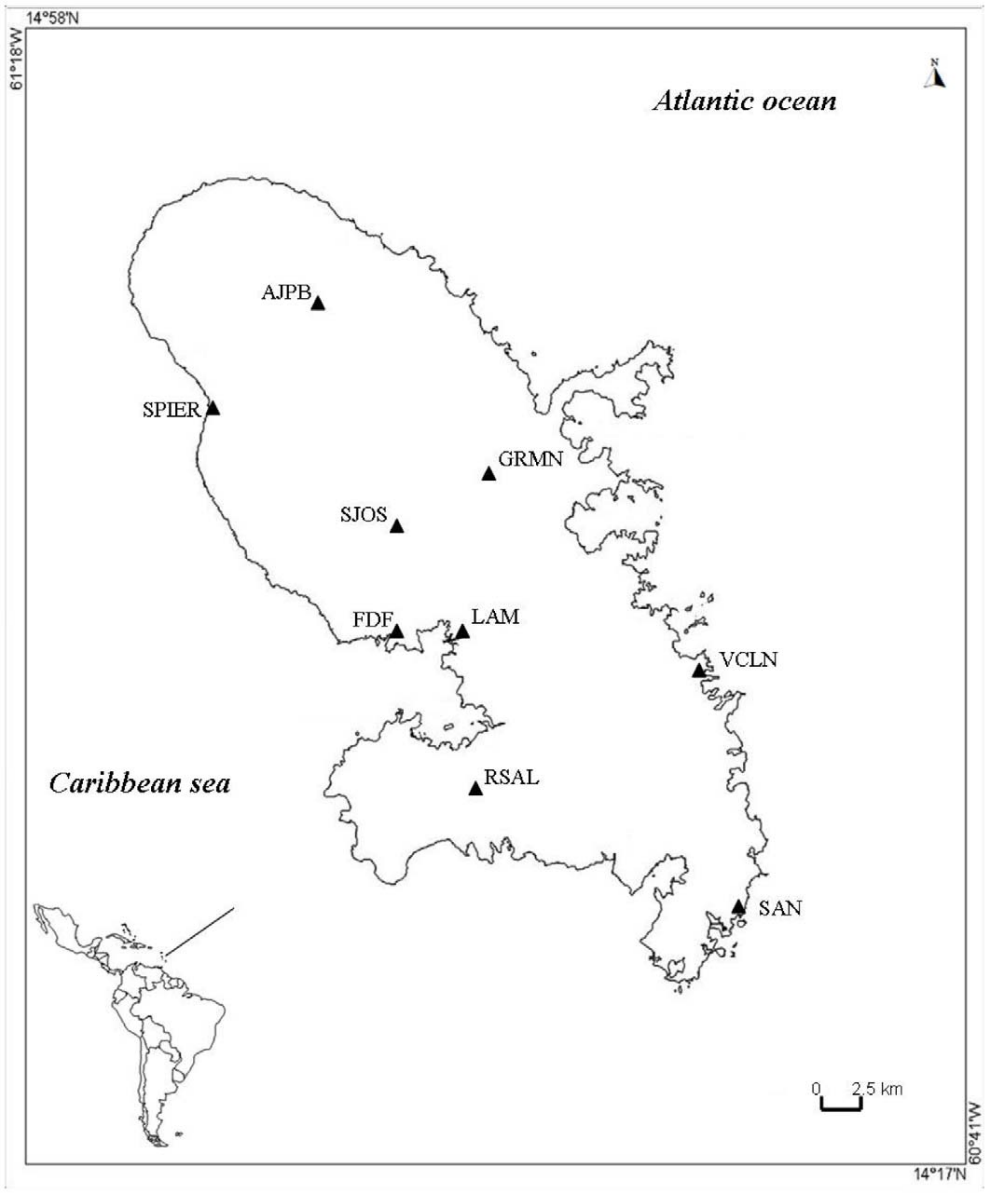
Location of *Ae. aegypti* populations sampling sites in Martinique. The samplings were made in the communities of Ajoupa-Bouillon, Saint Pierre, Gros Morne, Saint Joseph, Lamentin, Fort de France, Vauclin, Rivière Salée and Saint Anne respectively named AJPB, SPIER, GRMN, SJOS, LAM, FDF, VCLN, RSAL and SAN.

Two laboratory strains from French Polynesia (Bora-Bora) and Benin (SBE) and nine field-caught populations were used in the study. The two laboratory strains are susceptible to all insecticides and have been used as reference strains for resistance assays and as outgroups in order to gain comparative power and compensate for genetic diversity for gene expression analyses. *Ae. aegypti* was collected from individual houses as larvae or pupae in nine localities of Martinique in February 2009 (Figure 1). Populations were constituted from between 10 and 20 larval collection sites that were domestic breeding habitats. Mosquitoes from the same locality were pooled at the laboratory. Female were blood-fed on rabbit and larvae and adults obtained from the F1 progeny were used for bioassays, biochemical and molecular studies. The chosen populations covered most of the island ecotypes (coastal, mountainous, rural and urban). We also sampled populations from various mosquito habitats (urban, near agriculture, heavily treated, less treated, etc…). No specific permits were required for the described field studies. The mosquitoes were not collected from private land. We confirm that most of the sampling locations were not privately-owned. We asked the owners to sample in their back yards if needed. We also confirm that the locations were not protected in any way and that the field studies did not involve endangered or protected species.

### Larval and adult bioassays

Larval and adult bioassays were performed following WHO protocols [17], [18]. Larval bioassays were carried out using temephos (97.3%; Pestanal® Sigma-Aldrich, Riedel-de Haën, Germany) and the formulation of *Bti* Vectobac®12AS (1.2%, 1200 ITU/mg). Bioassays were performed using late third and early fourth-instar larvae of each population. Four replicates per concentration and 5 to 8 concentrations in the activity range of each insecticide were used (n = 100 per replicate per concentration). Larval mortality was recorded after 24 h of insecticide exposure.

Adult female mosquitoes of each population were used for the tarsal contact with treated filter papers in comparison with the Bora-Bora strain as described in Marcombe et al. [6]. Tests were run using filter papers treated with deltamethrin (0.05%) (100% [w/w]; AgrEVO, Herts, United Kingdom). Five batches of 20 non-blood fed females (2–5 days old; n = 100) were exposed to the insecticide during 60 minutes to estimate the knockdown effect (KD) of deltamethrin on each test population. For the control, females were exposed to papers impregnated with acetone mixed with silicone oil. Mortality was recorded 24 hours after the contact.

Percent mortality was corrected using Abbott's formula [19] when percent mortality from the control is greater than 5%. Mortality data were analysed by the log-probit method of Finney [20] using the Probit software of Raymond et al. [21]. Lethal concentrations (LC_50_ and LC_95_ for larvae) and knock-down time (KDT_50_ and KDT_95_ for adults) were calculated together with their 95% confidence intervals (95% CI). Adult mortality after 24 h exposure to deltamethrin was also recorded for each population. Populations from Martinique were considered as having different susceptibility to a given pesticide compared to the susceptible Bora-Bora strain when the ratio between their LC_50/95_ or KDT_50/95_ (resistance ratio: RR_50/95_) had confidence limits excluding the value of 1.

### Biochemical assays

P450 monooxygenases (P450s) level, carboxy/cholinesterases (CCEs) activities and Glutathione S-transferases (GSTs) activities were assayed from single 3 days-old F1 females (n = 47) according to the microplate methods described by Hemingway [22] and Brogdon [23]. Total protein quantification of each mosquito homogenates was performed using the Bio-Rad protein reagent kit with bovine serum albumin as the standard protein [24] in order to normalize enzyme activity levels by protein content. Statistical comparisons of detoxification enzyme levels between the susceptible strain Bora-Bora and other populations were assessed by using Mann Whitney's tests with Statistica software using a P value threshold of 0.05.

### *Kdr* genotyping

Total DNA of single female mosquitoes of each strain and Martinique populations (n = 32) was extracted using a CTAB protocol [25]. The region of the gene encoding the sodium channel where most *Kdr* mutations have been described [8], [26], [27] was amplified by PCR using Aed3 (5′ ACTACATCGAGAATGTGGATCG 3′) and Aed2A (5′ TTGTTGGTGTC GTTGTCGGCCGTCGG 3′) primers. This region covers exons 21 and 22 of the sodium channel gene allowing to detect the following *Kdr* mutations: I1011M, I1011V, V1016I and V1016G. After purification of the PCR products using the AMPure kit (Agencourt, Berverly, MA, USA), the BigDye terminator v3.1 kit (Applied Biosystems, Foster city, CA, USA) was used with the same primers for sequencing. Sequence reactions were purified using the CleanSEQ kit (Agencourt) and were then sequenced on an ABI Prism 3130xl analyzer (Applied Biosystems). The SeqScape software was used for sequence analysis. Hardy-Weinberg equilibrium was tested using the exact probability test [28].

### Enzymatic phenotyping of *Ache1*

The phenotypes of the acetylcholine esterase AChE1, encoded by the ace-1 gene, were investigated in each population (n = 24) using the previously described TDP test [29] adapted for *Ae. aegypti* with dichlorvos and propoxur concentrations of 3.10^−4^ M and 4.10^−4^ M, respectively. The TDP test allows discriminating all possible phenotypes containing the G119S, F290V and wild-type (susceptible) alleles.

### Constitutive transcription level of candidate detoxification genes

Transcription levels of 6 P450s (*CYP* genes), 2 P450-cofactors, the cytochrome-P450-reductase (*CPR* gene) and the cytochrome b5 (*CytB5* gene), 1 CCE (*CCEae3A*) and 3 GSTs genes were measured by real-time quantitative RT-qPCR in larvae and adult females of the 2 susceptible strains and the 9 field-caught populations. These genes were chosen because of their putative involvement in metabolic resistance to chemical insecticides [7], [13], [14], [30], [31]. Different batches of eggs from each strain/population were used to obtain 3 biological replicates of F1 individuals grown in standard insectary conditions. For each biological replicate, thirty 4^th^ stage larvae or thirty 4–6 days-old adult females were collected alive for RNA extraction using Trizol (Invitrogene). RNA extractions, reverse transcriptions and quantitative PCR analyses were performed as described in Marcombe et al [7]. Data analysis was performed according to the ΔΔC_T_ method taking into account PCR efficiency and using the genes encoding the ribosomal protein L8 (*AeRPL8*, GenBank accession number DQ440262) and R7 (*AeRPS7* GenBank accession number EAT38624.1) for normalization. Results were expressed as mean transcription ratios (±SE) between larvae or adults of the different populations and the susceptible Bora-Bora strain. Genes showing a transcription ratio higher than 2-fold and a P value<0.05 where considered significantly over-transcribed (Mann Whitney's test, N = 3).

### Relationship between insecticide resistance, transcription level of candidate genes and environmental factors

Potential relationship between insecticide resistance levels, gene transcription levels, *Kdr* mutation frequency and environmental factors characterizing each population were investigated through principal component analysis (PCA) across the nine populations of Martinique. Only candidate genes found over-transcribed in at least one population at any life stage were considered. The variables used and their respective ‘names’ were as follow: mean gene transcription ratio versus Bora-Bora strain (‘Gene_Lv’ for larvae or ‘Gene_Ad’ for adults), larval resistance to temephos (‘RR_50_ tem’), adult resistance to deltamethrin knock down effect (‘RR_50_ delta’), % adult surviving 24 h after deltamethrin exposure (‘Alive 24 h delta’), *Kdr* mutation frequency (‘V1016I’), insecticide pressure, agriculture and urbanization. Insecticides pressure was represented by two variables obtained from data provided by the vector control unit of Martinique. The first variable (‘Pulv’) represents the number of outdoor deltamethrin pulverization (thermal fogging) applications made routinely between 2006 and 2009 in each population area. The second variable (‘Int’) represents the number of specific interventions because of high entomological indices or dengue cases between 2006 and 2009, corresponding to larval treatment with *Bti* or temephos and deltamethrin pulverizations (outdoor thermal fogging and indoor spraying). The environment was described by five variables chosen for their putative role in the selection of insecticide resistance: sugar cane cultures (‘Sug’), bananas cultures (‘Ban’), other agricultural crops (‘Agri’), organochlorine pollution (‘OCPs Risk’, mainly chlordecone and lindane as described in Bocquene and Franco [12]) and urbanization (‘Urb’). Each environmental variable is expressed as the percentage of land surface of interest present in a circle of 2.5 km beam around each sampled sites (estimated average flying distance of mosquitoes). The satellites images used for the analysis where provided by the general council of Martinique (www.sig972.org. Accessed 2011 Jun 7). This percentage was obtained by pixel measurement using the software MESURIM (MESURIM Pro v3.4, with default parameter). PCAs were performed using R software [32]. As variables were not of the same scale, they were standardized (mean = 0 and standard deviation = 1) to avoid any distortion. Because no environmental variables were available for the susceptible strains, PCA was only performed on data from the nine Martinique populations (see Information S1 for all variables used for PCA).

## Results

### Larval and adult bioassays

Bioassays showed that the nine populations of Martinique were resistant to temephos and deltamethrin in comparison with the laboratory susceptible Bora-Bora strain and the susceptible strain SBE originating from Benin (Table 1). For temephos, RR_50_ ranged from 13-fold (SJOS) to 36-fold (GMRN) and RR_95_ ranged from 26-fold (SJOS) to 153-fold (VCLN). WHO tube tests with 0.05% deltamethrin on adult mosquitoes showed resistance to knockdown effect with RR_50_ ranging from 3.71-fold for RSAL population to 6.71-fold for SAN population. Mortality after 24 h deltamethrin exposure ranged from only 19% for AJPB population to 90% for the RSAL population while mortality in the susceptible strains reached 100%. For *Bti*, all Martinique populations showed low RRs comparatively to the susceptible Bora-Bora strain (maximum RR_50_ of 2.26-fold), indicating that all populations tested were mostly susceptible to *Bti*.

**Table 1:** Resistance status of *Aedes aegypti* populations of Martinique to *Bti*, temephos (larvae) and deltamethrin (adults).

Insecticide	Bti		Temephos		Deltamethrin		
Strain	RR_50_ (ci RR_50_)	RR_95_ (ci RR_95_)	RR_50_ (ci RR_50_)	RR_95_ (ci RR_95_)	RR_50_ (ci RR_50_)	RR_95_ (ci RR_95_)	Mortality(24 h)
Bora^1^							100%
SBE^1^	1,30 (1,26–1,29)	1,79 (1,58–1,83)	0,87 (0,84–0,87)	0,92 (0,92–0,95)	0,91 (1–0,92)	0,78 (0,81 – 0,7)	100%
AJPB	1,63 (1,58–1,71)	1,6 (1,37–1,97)	14,69 (13,33–16,67)	54,41 (42,5–73,75)	5,86 (5,21–6,93)	10,29 (8,15–15,81)	19%
SPIER	1,42 (1,46–1,41)	1,2 (1,19–1,3)	13,13 (12–15,15)	53,73 (41,61–74,53)	4 (3,86–4,21)	5,76 (5,3–6,86)	70%
GRMN	1,87 (1,91–1,89)	2,21 (2,03–2,66)	35,94 (29,67–44,85)	149,49 (102,32–216,56)	5,36 (4,86–6,14)	6,95 (5,9–9,62)	64%
SJOS	1,02 (0,89–1,18)	0,73 (0,57–0,96)	12,81 (10,67–16,06)	26,27 (16,25–42,34)	5,79 (5,21–7)	8,48 (6,8–13,38)	29%
LAM	1,87 (1,77–2,03)	1,9 (1,42–2,66)	31,56 (28,33–35,76)	138,64 (112,68–176,25)	5,64 (5–6,79)	8,05 (6,45–12,81)	55%
FDF	1,81 (1,74–1,92)	1,95 (1,55–2,58)	14,69 (13,67–16,36)	35,76 (29,64–45)	6,43 (5,5–8,64)	9,43 (7–18,19)	23%
VCLN	1,27 (1,28–1,27)	1,16 (1,13–1,26)	27,5 (24,67–31,52)	152,88 (116,79–211,41)	5,71 (5,14–6,79)	7,14 (5,95–10,52)	20%
RSAL	1,4 (1,42–1,39)	1,2 (1,18–1,3)	28,75 (26,33–32,73)	81,69 (64,82–110)	3,71 (3,57–3,79)	4,76 (4,25–4,71)	90%
SAN	2,26 (2,32–2,29)	2,34 (2,12–2,88)	19,06 (17,67–21,52)	55,93 (45–72,66)	6,71 (5,71–8,57)	12,19 (9,05–21,52)	42%

1 Susceptible reference strains. LC_50_ and LC_95_ in mg/liter for the Bora strain were 0.062 and 0.14 with *Bti*, 0.0032 and 0.0059 with temephos. KDT_50_ and KDT_95_ were 14 and 21 min with deltamethrin. ci: confidence interval.

### Detoxification enzyme levels

Detoxification enzyme activities were estimated for each population at the adult stage (Table 2). For all enzyme families, activities measured in the susceptible strain from Benin (SBE) were lower than in the susceptible Bora-Bora. P450s level were significantly higher than in the Bora-Bora strain (Mann-Whitney's test) in all Martinique populations except RSAL. In comparison with the Bora-Bora strain, α-CCEs activities were significantly higher for SJOS, VCLN and SAN populations while ß-CCEs activity was only significantly higher in the SAN population. GSTs activities were significantly elevated in AJPB, SPIER, VCLN, RSAL and SAN populations in comparison with Bora-Bora strain.

**Table 2:** Detoxification enzyme activities in adults of the populations of Martinique and the laboratory strains: cytochrome P450 monooxygenases (P450s; nmol P450 U/mg protein), Esterase (α and β-CCEs; α/β-Naphtol/min/mg protein) and Glutathione-S-transferases (GSTs; GSH/min/mg protein).

Strain	n	P450 (± sd)	Esterases α (± sd)	Esterases β (± sd)	GST (± sd)
Bora^1^	47	0,039 (±0,0046)	0,108 (±0,0153)	0,077 (±0,0169)	0,020 (±0,0485)
SBE^1^	47	0,028 (±0,0032)*	0,081 (±0,0092)*	0,071 (±0,0131)	0,015 (±0,0902)
AJPB	47	0,054 (±0,0059)*	0,110 (±0,0095)	0,081 (±0,0138)	0,072 (±0,0406)*
SPIER	47	0,053 (±0,0112)*	0,104 (±0,0263)	0,074 (±0,0195)	0,037 (±0,0429)*
GRMN	47	0,043 (±0,0049)*	0,103 (±0,0144)	0,061 (±0,0145)	-
SJOS	47	0,045 (±0,007)*	0,146 (±0,0308)*	0,085 (±0,0211)	-
LAM	47	0,056 (±0,0062)*	0,111 (±0,0139)	0,074 (±0,0153)	0,021 (±0,0559)
FDF	47	0,050 (±0,0044)*	0,114 (±0,0118)	0,073 (±0,0129)	0,039 (±0,0498)
VCLN	47	0,054 (±0,0081)*	0,120 (±0,0189)*	0,072 (±0,0148)	0,038 (±0,0492)*
RSAL	47	0,040 (±0,0087)	0,104 (±0,0227)	0,064 (±0,0173)	0,064 (±0,0452)*
SAN	47	0,053 (±0,0079)*	0,125 (±0,0127)*	0,098 (±0,0188)*	0,051 (±0,0463)*

1 Susceptible reference strains.

*: Values significantly different in field-caught populations comparatively to the Bora laboratory strain (Mann and Whitney's test, p<0.05). sd: standard deviation.

### *Kdr* genotyping

Sequencing of the voltage-gated sodium channel gene from single mosquitoes revealed the presence of the *Kdr* mutation at position 1016 (GTA to ATA) leading to the replacement of a valine by an isoleucine (V1016I) at a high allelic frequency for all of Martinique populations (f[R] ranged from 0.87 to 0.97, Table 3). All the populations were at Hardy-Weinberg equilibrium (Exact test, *P*>0.05). No other *Kdr* resistant allele was detected in these populations. No *Kdr* mutation was found in the two susceptible strains Bora-Bora and SBE.

**Table 3:** Frequency of the V1016I *kdr* mutation in the populations of Martinique and the two reference strains.

Strain	n	RR	RS	SS	R frequency
Bora^1^	31	0	0	31	0,00
SBE^1^	31	0	0	31	0,00
AJPB	32	25	7	0	0,89
SPIER	31	24	6	1	0,87
GRMN	32	26	6	0	0,91
SJOS	32	24	8	0	0,88
LAM	31	29	2	0	0,97
FDF	32	28	4	0	0,94
VCLN	32	26	6	0	0,91
RSAL	32	25	7	0	0,89
SAN	32	27	5	0	0,92

1 Susceptible reference strains; n: number of females tested; RR: number of homozygous resistant individuals; SS: number of homozygous susceptible individuals; RS: number of heterozygous individuals; R frequency: Resistance allele frequency in the population.

### Enzymatic phenotyping of *AChE1*

All mosquito test populations from Martinique showed similar percentages of AChE inhibition with dichlorvos and propoxur compared to the susceptible Bora-Bora strain excepted for RSAL and VCLN (only for dichlorvos for this latter). However, the differences were not strong enough to suspect the presence of insensitive AChE in the populations tested (Table 4).

**Table 4:** Mean percentage of the AChE inhibition by dichlorvos and propoxur in the laboratory strains and the populations of Martinique.

Strain	n	dichlorvos	± sd	propoxur	± sd
Bora^1^	80	74,99	7,09	80,13	4,45
SBE^1^	24	70.77	3.44	76.48	2.91
AJPB	24	76,95	4,26	80,99	3,02
SPIER	24	83,74	7,24	83,67	4,22
GRMN	24	73,95	5,68	81,99	4,26
SJOS	24	77,21	7,70	82,43	4,70
LAM	24	80,05	3,60	82,42	3,73
FDF	24	77,54	4,61	82,56	3,27
VCLN	24	71,20*	3,97	82,23	4,97
RSAL	24	65,52*	3,75	75,20*	3,03
SAN	24	76,80	3,59	82,74	3,50

1 Susceptible reference strains.

*: Values significantly lower in field populations comparatively to Bora strain (Mann and Whitney's test, p<0.05). sd: standard deviation.

### Constitutive transcription level of candidate detoxification genes

**Figure 2:**
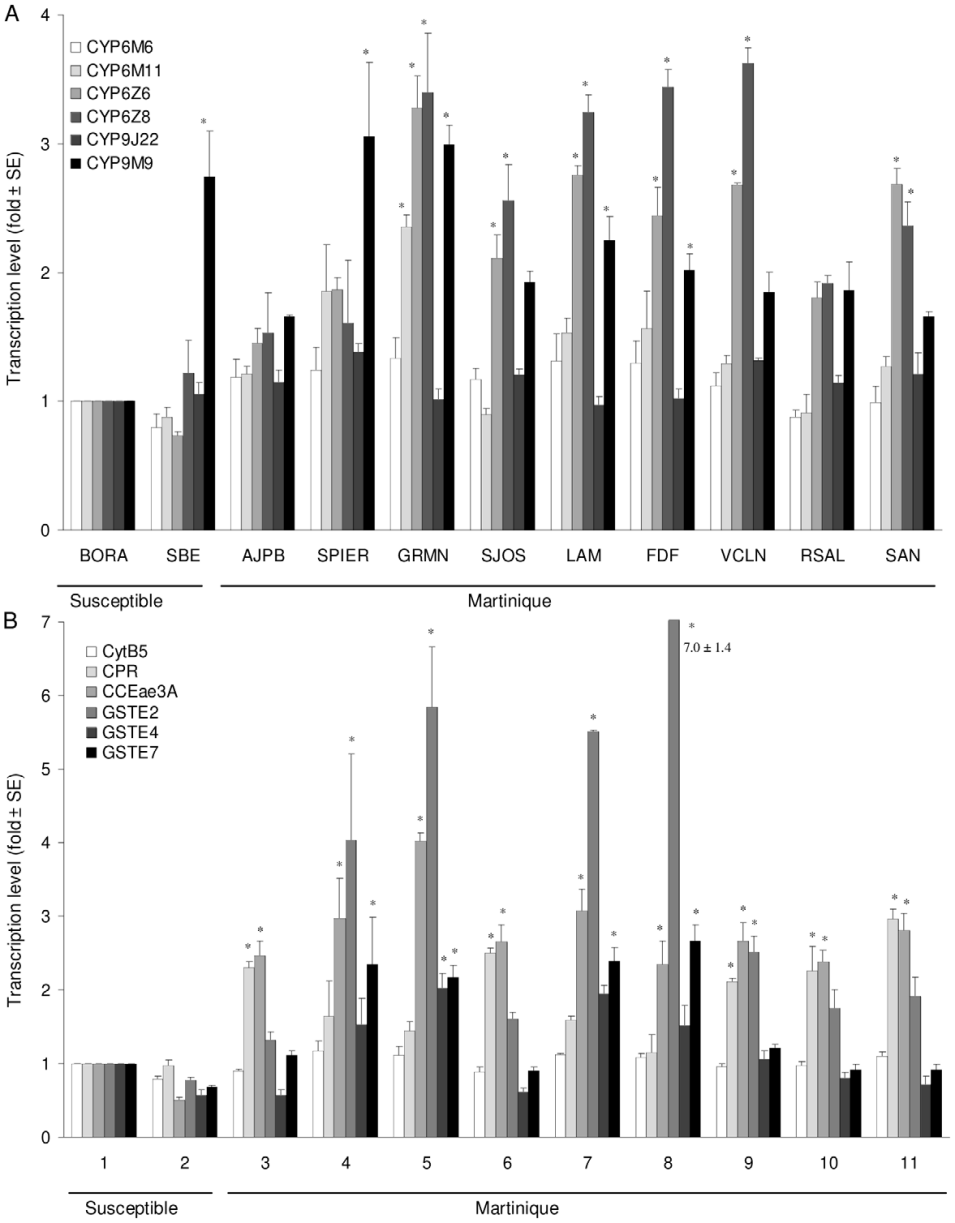
Larval transcription levels of (A) 6 cytochrome-P450-monooxygenases and (B) 2 P450-cofactors, 3 GST and 1 CCE genes estimated in the populations of Martinique and the susceptible SBE strain relative to the susceptible Bora-Bora strain. Transcription ratios obtained from real-time quantitative RT-PCR were normalized with the 2 housekeeping genes *AeRPL8* and *AeRPS7* and shown as mean value (± SD) over 3 independent biological replicates. Genes significantly over-transcribed (transcription ratio >2-fold and P value<0.05) are indicated by stars.

Transcription profiles of twelve candidate detoxification genes potentially involved in metabolic resistance to insecticides were compared between susceptible strains and Martinique test populations at the larval and adult stages. Genes with transcription ratio over 2-fold and a P value<0.05 were considered significantly over-transcribed. In larvae (Figure 2), the P450 genes *CYP6Z6* and *CYP6Z8* were both over-transcribed in 6 populations of Martinique (GRMN, SJOS, LAM, FDF, VCLN and SAN) compared to the susceptible strains (Figure 2A). The gene *CYP6M11* was over-transcribed in the GRMN population. *CYP9M9* was over-transcribed in SPIER, GRMN, LAM, FDF Martinique populations but also in the susceptible strain SBE. Among other genes (Figure 2B), the P450 co-factor *CPR* was over-transcribed in the AJPB, SJOS, VCLN, RSAL and SAN populations. The esterase gene *CCEae3A* was over-transcribed in all Martinique populations. Among GST genes, *GSTE2* and *GSTE7* were both over-transcribed in SPIER, GRMN, LAM and FDF populations with a higher over-transcription of *GSTE2* (up to 7-fold).

**Figure 3:**
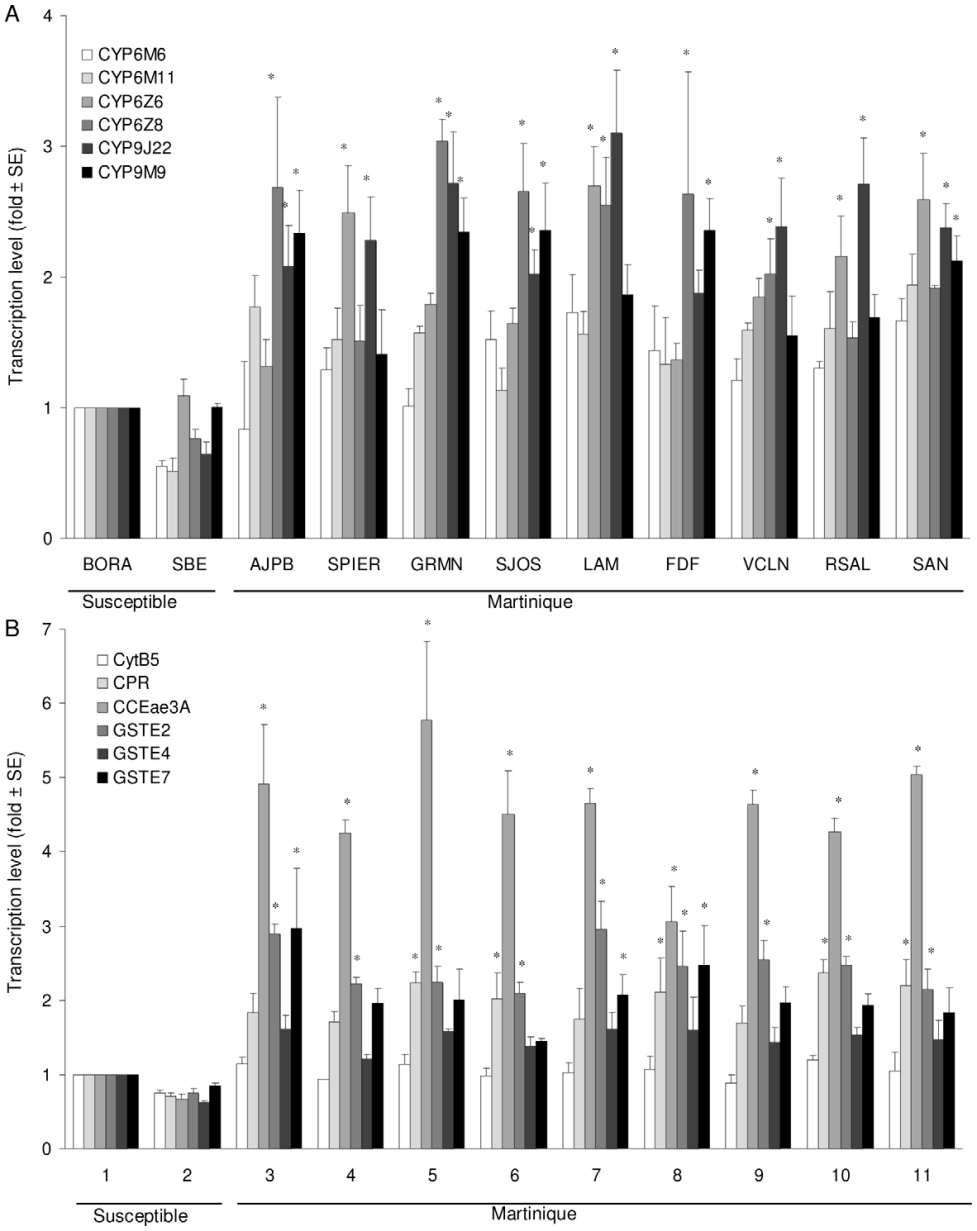
Adult transcription levels of (A) 6 cytochrome-P450-monooxygenases and (B) 2 P450-cofactors, 3 GST and 1 CCE genes estimated in the populations of Martinique and the susceptible SBE strain relative to the susceptible Bora-Bora strain. Transcription ratios obtained from real-time quantitative RT-PCR were normalized with the 2 housekeeping genes *AeRPL8* and *AeRPS7* and shown as mean value (± SD) over 3 independent biological replicates. Genes significantly over-transcribed (transcription ratio >2-fold and P value<0.05) are indicated by stars.

In adults, four different P450 genes were over-transcribed in Martinique populations (Figure 3A). The genes *CYP6Z6* and *CYP6Z8* were over-transcribed in SPIER, LAM, RSAL, SAN and AJPB, GRMN, SJOS, LAM, FDF, VCLN respectively. *CYP9J22* was over-transcribed in all Martinique populations except FDF while *CYP9M9* was over-transcribed in AJPB, GRMN, SJOS, FDF and SAN. No significant differences of transcription level were observed for *CYP6M6* and *CYP6M11* compared to the susceptible strains. Among the two P450-cofactor genes, only the *CPR* was over-transcribed in the GRMN, SJOS, FDF, RSAL and SAN populations (Figure 3B). As in larvae, the *CCEae3A* gene was over-transcribed in all Martinique populations. Among GSTs, *GSTE2* was over-transcribed in all Martinique populations whereas *GSTE7* was over-transcribed in four populations only (AJPB, GRMN, LAM and FDF).

### Relationship between insecticide resistance, transcription levels of candidate genes and environmental factors

**Figure 4:**
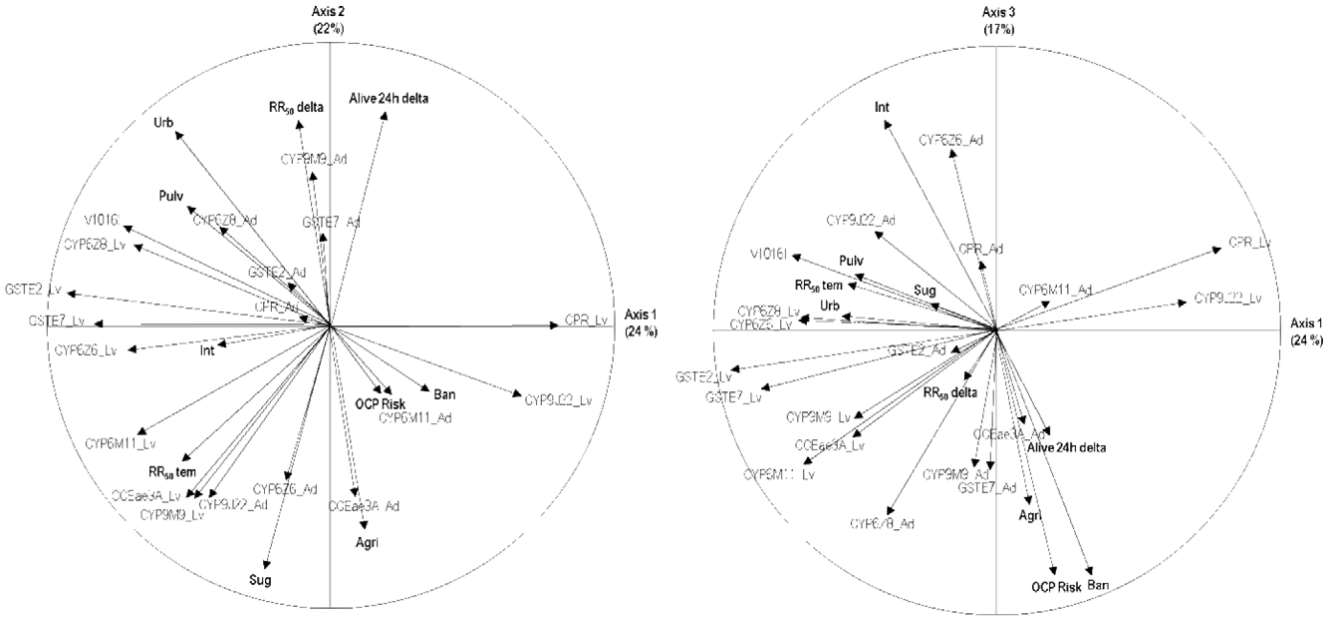
Graphical representation of the 29 variables on the three first axes of the principal component analysis (PCA). Percent of starting information represented by each PCA axis are indicated. Variables related to insecticide resistance levels and environment are shown in bold. V1016I: % V1016I *Kdr* mutation; RR_50_ delta: RR_50_ to deltamethrin knock down effect; Alive 24 h delta: % alive adult mosquitoes 24 hours after deltamethrin exposure ; RR_50_ tem: larval RR_50_ to temephos; Pulv: deltamethrin thermal fogging application events from 2006 to 2009; Int: Insecticide treatments intervention events from 2006 to 2009; Urb, Agri, Ban, Sug: % land surface of urbanization, agriculture, bananas and sugar culture respectively around each population; OCPs Risk: organochlorine pollution (mainly chlordecone and lindane).

Principal Component Analysis (PCAs) was performed on all Martinique populations with 29 variables including insecticide resistance levels, larval and adult transcription ratios of candidate genes and environmental variables. Relations among variables across all Martinique populations are presented in Figure 4 and detailed results are shown in Information S2. The first three PCA axes resumed 63% of the starting information with 24%, 22% and 17% respectively. Larval resistance to temephos was mainly represented on the two first PCA axes and strongly positively correlated to sugar cane culture and larval over-transcription of *CCEae3A*, *CYP6M11*, *CYP9M9*. Larval resistance to temephos was negatively correlated to adult deltamethrin resistance. Adult deltamethrin resistance was mainly represented by the second PCA axis and positively correlated to urbanization, deltamethrin thermal fogging application and the adult over-transcription of *CYP9M9*, *GSTE7* but negatively correlated to agriculture, sugar cane culture and the adult over-transcription of *CYP6Z6* and *CCEae3A*. Deltamethrin application was positively correlated with *Kdr* mutation frequency. Variables related to agriculture were mainly represented by the second and third PCA axes and positively correlated together. Sugar cane culture and in a lesser extent other agriculture variables were positively correlated to temephos resistance but not to deltamethrin resistance. Finally, one should note that several groups of genes showed a strong correlation of their transcription levels across the three first PCA axes such as *CYP6Z6*, *CYP6Z8*, *GSTE7* and *GSTE2* in larvae; *CCEae3A*, *CYP6M11* and *CYP9M9* in larvae; *CPR* and *CYP9J22* in larvae or *CYP9M9* and *GSTE7* in adults.

## Discussion

Toxicological results showed that all *Ae. aegypti* populations of Martinique were resistant to temephos and deltamethrin but mostly susceptible to *Bti*. For *Bti*, although a slight resistance to *Bti* toxins could not be excluded [33], the significant resistant ratios (RRs) measured for Martinique populations compared to the Bora-Bora test population may underline a different genetic background between field populations and laboratory test population. As *Bti* remains the main insecticide available for larval treatments, the low level of resistance is encouraging for future vector control in Martinique.

Among target-site mutations conferring resistance to chemical insecticides, only the V1016I *Kdr* mutation was found in Martinique populations, confirming the results previously obtained by Saavedra-Rodriguez et al. [9] in South America and Caribbean. High frequency of the V1016I *Kdr* mutation was observed among the nine populations, indicating that deltamethrin resistance is partly associated with target site mutation. Donnelly et al. [34] pointed out a strong causal relationship between *Kdr* genotype and susceptibility to DDT and pyrethroids in many mosquito species, including *Ae. aegypti*. Saavedra-Rodriguez et al. [35] showed the beneficial effect of the V1016I *Kdr* mutation regarding knock-down time, recovery and survival rate of *Ae. aegypti* adults exposed to pyrethroids. Our study revealed a wide range of survival rates after deltamethrin exposure (20% to 90%) while the V1016I *Kdr* mutation was almost fixed in all sampled populations (f≥0.87), suggesting that other resistance mechanisms may involved in mosquito test population from Martinique. One should note that two novel mutations in the sodium channel gene (mutation F1552C and F1534C) linked to pyrethroid and DDT resistance has been recently found in *Ae. aegypti* in Thailand [36] and in the Cayman Islands [37], suggesting that other undiscovered target site mutations might also contribute to pyrethroid resistance.

Enzymatic phenotyping of AChE1 did not allow demonstrating the presence of the G119S and F290V mutations in organophosphate resistance in mosquito test population from Martinique. However, the RSAL population presented a slightly lower inhibition rates for dichlorvos and propoxur compared to other populations. The sequencing of the *Ace.1* gene in this population should confirm the absence of this mutation in Martinique. Unless other mutations are present elsewhere in this gene, our results showed that organophosphate resistance is mainly due to metabolic mechanisms.

By quantitative trait loci (QTL) mapping, Saavedra-Rodriguez et al. [35] confirmed that genes coding for detoxification enzymes play a significant role in pyrethroid resistance in *Ae. aegypti*. Biochemical assays on adults showed that deltamethrin resistance seemed to be associated with higher P450 levels confirming previous results obtained by Marcombe et al. [7] on a single Martinique population. In the present study, the 9 populations tested showed significantly higher P450s activities except in the RSAL population which was also the less resistant to deltamethrin. Five populations also showed higher GST activities compared to the susceptible Bora-Bora strain. As observed by Rodriguez et al. in Cuba [38], GST detoxification enzymes may be involved in deltamethrin resistance in Martinique, although no *Ae. aegypti* GST has yet been shown to metabolize pyrethroids or their metabolites. Four populations presented higher activities of α-CCEs (Table 2) which have been previously involved in organophosphate resistance [39]. Elevated esterase activities were also observed by Marcombe et al. [7] who showed higher activities of CCEs and in a lesser extent P450s in *Ae. aegypti* larvae from Martinique.

At the molecular level, metabolic resistance of Martinique populations through over-expression of detoxification enzymes was investigated by quantitative RT-PCR on 12 candidate genes. Our results showed that several candidate genes were over-transcribed in Martinique populations comparatively to the susceptible strains. Among them, *CYP6Z6*, *CYP6Z8*, *GSTE7* and *CPR* seemed to be over-transcribed to a similar extent at both life stages, while others showed a more pronounced over-transcription in adults (*CYP9J22* and *CCEae3A*) or larvae (*GSTE2*). Such life-stage specific over-transcription patterns suggest that particular enzymes might be more specifically involved in resistance to chemical insecticides during a particular life stage [40], [41].

Over-transcription of genes encoding P450s has been frequently associated with metabolic-based insecticide resistance in insects [42]. In mosquitoes, the *CYP6Z* subfamily has been previously associated with response to pyrethroid, carbamate and organochlorine insecticides [43], [44], [45], [46]. In *Ae. aegypti*, *CYP6Z9* has been found 4-fold over-transcribed in a permethrin-resistant mosquito population collected in Northern Thailand [30] and *CYP6Z8* was also identified as inducible by permethrin and other pollutants [15], [41]. The over-transcription of *CYP6Z6* and *CYP6Z8* in most Martinique populations confirms the possible involvement of *Ae. aegypti CYP6Zs* in insecticide resistance in Martinique. The P450 gene *CYP9M9* was found over-transcribed in several Martinique populations at both life stages. This gene was found to be inducible by permethrin, temephos and others pollutants [13], [14]. However, in the present study, *CYP9M9* was also over-transcribed in larvae of the susceptible strain SBE from Benin and showed important variations between populations, suggesting that this gene may not have a major role in resistance. Conversely, the repeated over-transcription of *CYP9J22* in adults is in agreement with results obtained by Marcombe et al. [7] and suggests a significant role of this gene in resistance. Recently, the capacity of other *Ae. aegypti CYP9Js* to metabolize pyrethroids was validated by heterologous expression followed by *in-vitro* insecticide metabolism assays (M. Paine, personal communication), confirming the involvement of this P450 subfamily in insecticide resistance. Finally, Lycett *et al.* [47] showed that the silencing of the P450 electron donor cytochrome P450 reductase (*CPR*) causes an increased susceptibility to permethrin in *An. gambiae*. Therefore, the recurrent elevated transcription level of the *CPR* gene at both life stages supports the major role of the P450 detoxification system in metabolic resistance mechanisms in Martinique.

The over-transcription of the gene *GSTE2* is of particular interest since the associated enzyme has been shown to metabolize DDT in *An. gambiae* and *Ae. aegypti* [31], [48]. *GSTE2* was also found over-transcribed in a DDT- and pyrethroid-resistant mosquito population from Thailand [31]. The intensive use of DDT and other organochlorines may be at the origin of the selection of the constitutive over-transcription of *GSTE2* in Martinique. The gene *GSTE7* was also found over-transcribed in several Martinique populations, and in several pyrethroid resistant *Ae. aegypti* strain from Thailand [30]. Recent studies performed in our laboratory showed that this enzyme show a GSH conjugation activity and is able to bind several insecticides and pollutants such as DDT, pyrethroids and temephos (A. Chandor-Proust and J.P. David personal communication).

The esterase gene *CCEae3A* was constitutively over-transcribed in all Martinique populations at both life stages and highly correlated with temephos resistance. The massive use of temephos during decades in Martinique may have selected for a CCE based metabolic resistance and particularly for the *CCEae3A* gene. Further work on the phenotypic expression of this candidate gene in *Aedes* would be required as esterases are known to play an important role in OPs resistance in mosquitoes [49], [50], [51].

The principal component analysis of 29 variables characterizing the 9 populations of Martinique underlined potential relationships between variables. Negative correlation between resistance to temephos and to deltamethrin suggests not only the absence of cross-resistance between the two insecticides but also that both resistances are submitted to different selection pressures in Martinique. The absence of correlation between deltamethrin resistance and agriculture variables may indicate that agricultural pesticides do not play a major role on adult deltamethrin resistance in Martinique or eventually that this impact is too homogenous across populations to be highlighted by our analysis. A significant relationship appeared between urbanization, deltamethrin application and *Kdr* mutation frequency possibly associated to the selection pressure applied in urban zones by deltamethrin treatments. Recent studies (Marcombe et al unpublished) showed a surprising strong genetic differentiation of the *Aedes* populations of Martinique. Larval or adult treatments especially in urban zone may have an influence on the resistance selection, the effective population size (bottleneck) and on the population structure. However, the correlation between *Kdr* mutation frequency and deltamethrin resistance was not so significant, confirming that metabolic resistance mechanisms play a significant role in deltamethrin resistance and that other sources of selection exist like household insecticides. The agriculture and sugar cane culture variables were correlated with larval resistance to temephos suggesting a potential role of agriculture in the selection of organophosphate resistance in Martinique. Many studies showed that agricultural practices such as cotton or vegetable culture may have an essential role in the selection for DDT and pyrethroid resistance, especially for the main malaria vector *Anopheles gambiae* in Africa [52], [53], [54]. The fact that *Ae. aegypti* is mainly associated to urban and peri-urban environments explain the lesser impact of agricultural practices on this resistance pattern.

From the present study, it could be hypothesized that deltamethrin resistance in Martinique results from the combination of the presence of the V1016I *Kdr* mutation and the over-production of detoxification enzymes such as with CYP6Zs and GSTs participating in phase I and phase II detoxification steps respectively. At the larval stage an interesting positive correlation was observed between *CCEae3A* transcription level and temephos resistance, confirming the potential role of this gene in metabolic resistance of this insecticide. However, such correlation was also observed with the P450 genes *CYP6M11* and *CYP9M9* suggesting that multiple detoxification enzyme families may be involved in temephos resistance.

This study showed that resistance to chemical insecticides is multiple and widely distributed among *Ae. aegypti* Martinique populations.

Several detoxification genes such as P450s belonging to the *CYP6Z, CYP9J or CYP6M* subfamilies, *epsilon GSTs* or the esterase *CCEae3A* represent good candidates for further functional validation. Although it cannot be excluded that other genes are involved in insecticide resistance, gene silencing and heterologous expression approaches will provide more evidences of their potential role in metabolic resistance.

The role of environmental factors such as agriculture in *Ae. aegypti* resistance to insecticide in Martinique was preliminarily investigated through a multivariate analysis. Additional population genetic and genomic studies are currently performed on *Ae. aegypti* Martinique populations. These studies should bring more information about the genetic structure of Martinique populations and eventually point out specific genetic region under selection. A better understanding of the genetic basis of insecticide resistance is essential to optimize vector control strategies.

## Supporting Information

Information S1 **Variables used for PCA analyses.**
(XLS)

Information S2 **Detailed PCA analysis.**
(XLS)
